# Recurrent Amplification at 13q34 Targets at *CUL4A*, *IRS2*, and *TFDP1* As an Independent Adverse Prognosticator in Intrahepatic Cholangiocarcinoma

**DOI:** 10.1371/journal.pone.0145388

**Published:** 2015-12-18

**Authors:** Ting-Ting Liu, Huey-Ling You, Shao-Wen Weng, Yu-Ching Wei, Hock-Liew Eng, Wan-Ting Huang

**Affiliations:** 1 Department of Pathology, Kaohsiung Chang Gung Memorial Hospital and Chang Gung University College of Medicine, Kaohsiung, Taiwan; 2 Department of laboratory Medicine, Kaohsiung Chang Gung Memorial Hospital and Chang Gung University College of Medicine, Kaohsiung, Taiwan; 3 Department of Internal Medicine, Kaohsiung Chang Gung Memorial Hospital and Chang Gung University College of Medicine, Kaohsiung, Taiwan; 4 Department of Medical Laboratory Sciences and Biotechnology, Fooyin University, Kaohsiung, Taiwan; 5 Department of Pathology, Kaohsiung Municipal Ta-Tung Hospital, Kaohsiung Medical University Hospital, Kaohsiung, Taiwan; Wayne State University School of Medicine, UNITED STATES

## Abstract

Amplification of genes at 13q34 has been reported to be associated with tumor proliferation and progression in diverse types of cancers. However, its role in intrahepatic cholangiocarcinoma (iCCA) has yet to be explored. We examined two iCCA cell lines and 86 cases of intrahepatic cholangiocarcinoma to analyze copy number of three target genes, including *cullin 4A (CUL4A)*, *insulin receptor substrate 2 (IRS2)*, and *transcription factor Dp-1 (TFDP1)* at 13q34 by quantitative real-time polymerase chain reaction. The cell lines and all tumor samples were used to test the relationship between copy number (CN) alterations and protein expression by western blotting and immunohistochemical assays, respectively. *IRS2* was introduced, and each target gene was silenced in cell lines. The mobility potential of cells was compared in the basal condition and after manipulation using cell migration and invasion assays. CN alterations correlated with protein expression levels. The SNU1079 cell line containing deletions of the target genes demonstrated decreased protein expression levels and significantly lower numbers of migratory and invasive cells, as opposed to the RBE cell line, which does not contain CN alterations. Overexpression of IRS2 by introducing *IRS2* in SUN1079 cells increased the mobility potential. In contrast, silencing each target gene showed a trend or statistical significance toward inhibition of migratory and invasive capacities in RBE cells. In tumor samples, the amplification of each of these genes was associated with poor disease-free survival. Twelve cases (13.9%) demonstrated copy numbers > 4 for all three genes tested (*CUL4A*, *IRS2*, and *TFDP1*), and showed a significant difference in disease-free survival by both univariate and multivariate survival analyses (hazard ratio, 2.69; 95% confidence interval, 1.23 to 5.88; *P* = 0.013). Our data demonstrate that amplification of genes at 13q34 plays an oncogenic role in iCCA featuring adverse disease-free survival, which may provide new directions for targeted therapy.

## Introduction

Gene copy number (CN) alterations are associated with a number of human diseases [1]. Amplification is one mechanism for overexpression of oncogenes, a critical step in cancer development and progression. Activation mutations, rather than amplification, of genes such as *Kirsten rat sarcoma viral oncogene homolog* (*KRAS*), *isocitrate dehydrogenase 1* (*IDH1*), *IDH2*, *B-Raf proto-oncogene*, *serine/threonine kinase* (*BRAF*), and *epidermal growth factor receptor* (*EGFR*), have been shown to have a potential impact on the prognosis of intrahepatic cholangiocarcinoma (iCCA) [2]. Knowledge of the effect of gene amplification is limited. Some whole-genome CN analyses of iCCA revealed frequent CN gains on chromosome 1q, 5p, 7p, 8q, 17q and 20q [3,4]. Chromosome 1q gains and deoxyribonucleic acid (DNA) amplifications at 11q13.2 were associated with worse outcome [3,4]. In one small-scale study, cyclin D1 gene was found to be amplified in five out of 20 patients associated with poor histological differentiation [5]. Ukita et al. demonstrated 100% amplification of *human epidermal growth factor receptor 2* (*HER2*), correlated with overexpression of the c-erbB-2 protein, in 22 archival iCCA cases [6].

DNA amplification at 13q34 has been observed in diverse types of cancers, for which several candidate target genes have been identified. These genes include: *transcription factor Dp-1 (TFDP1)*, *cullin 4A* (*CUL4A)*, and *cell division cycle 16* (*CDC16)* identified in hepatocellular carcinoma (HCC) [7], breast cancer [8], and lung cancer [9]; and *insulin receptor substrate 2* (*IRS2)* present in colorectal cancer [10]. CUL4A overexpression has been reported to be associated with migration, invasion, epithelial-mesenchymal transition and disease progression [11–13]. The role and frequency of gene CN alterations at 13q34 in iCCA has yet to be investigated. In our previous genome-wide study of combined HCC and cholangiocarcinoma, we found that amplification of 13q34 was present in the cholangiocarcinoma rather than the HCC component [14].

In this present study, we first examined CN alteration, protein expression and mobility potential in two iCCA cell lines, and then looked at 86 cases of iCCA from a single institute to examine three target genes at 13q34. CN alterations of target genes were determined by quantitative real-time polymerase chain reaction (qPCR), and correlated with clinicopathologic features. We therefore aimed to examine (1) the frequency of 13q34 amplification and (2) whether this molecular aberration correlates with protein overexpression and disease progression in iCCA.

## Materials and Methods

### Specimens and cell lines

The iCCA cell lines, SNU1079 and RBE, were purchased from the Korean Cell Line Bank (Seoul, South Korea) and the Riken BRC Cell Bank (Koyadai, Japan), respectively. Tumor cell lines were cultured in Roswell Park Memorial Institute (RPMI) medium (Gibco-BRL, Carlsbad, CA, USA) supplemented with 10% fetal bovine serum (FBS, Gibco-BRL) and antibiotic-antimycotic (100 U/ml penicillin, 100 μg/ml streptomycin, and 25 μg/ml amphotericin) (Life-technologies, Grand Island, NY, USA). Cells were grown at 37°C in a humidified incubator containing 5% CO_2_. Eighty-six cases of formalin-fixed, paraffin-embedded tumor and non-tumor samples from 2003 to 2012 were obtained from the files of the Department of Pathology, Chang Gung Memorial Hospital at Kaohsiung, Taiwan. The medical records associated with the samples were available and were carefully reviewed. Survival time was defined as the time period between the date of diagnosis and the date of death or the patient’s last follow-up. The hematoxylin and eosin-stained sections obtained at the time of diagnosis and repeats were reviewed. The American Joint Committee on Cancer (AJCC) 7th edition staging system was adopted for the staging of iCCA. The study was approved by the institutional review board, in accordance with the Helsinki Declaration (IRB 103-0818C). For patients dying of the disease, no informed consent was available; therefore all samples and medical data used in this study have been irreversibly anonymized. The other patients have given their written informed consent.

### DNA extraction and copy number evaluation

qPCR of target genes was performed on extracted DNA to determine CNs in both test samples and cell lines, as previously described [14]. The respective non-tumor liver tissues of patients and peripheral blood nuclear cells (reference samples) of three healthy individuals were included as control samples. Commercially available CN assays for the FAM-labeled probe TFDP1 (ABI assay ID: Hs02418979), CUL4A (ABI assay ID: Hs01869233), IRS2 (ABI assay ID: Hs03046114), and VIC-labeled probe RNase P (ABI Part Number: 4403326) were obtained from Applied Biosystems (Applied Biosystems, Foster City, CA). RNase P was used as the endogenous control. Assays were conducted in triplicate. The CN (q) of the target genes was determined using the comparative quantitative threshold cycle (ΔΔCt) method, where ΔΔCt = (Ct of target gene, test sample − Ct of RNase P, test sample) − (average Ct of target gene, reference samples − average Ct of RNase P, reference samples) and the 2 × 2^− ΔΔCt^ formula. For tumor samples, the relative CN (Q) was calculated using the following equation:

$$\text{Q}=2\frac{\text{qT}}{\text{qN}}$$

Where qT is the CN of target genes in the tumor tissue, and qN is the CN of target genes in the non-tumor section. For statistical analysis, cases were classified into three groups according to the Q cut-off values, which were set at 4.0 to define the gain; and < 2 to define the loss.

### Western blot analysis

RBE and SNU1079 cells reaching confluence in culture were harvested and washed with ice-cold phosphate-buffered saline (PBS). Cells were then lysed with lysis buffer (20 mM HEPES, 20% glycerol, 1.5 mM MgCl_2_, 0.2 mM EDTA, 0.1% Triton X-100, 500 mM NaCl, 1 mM DTT, and 1 μg/ml of the protease inhibitors (pepstatin, leupeptin, and aprotinin). The extracts were centrifuged at 14000 × *g* for 10 min at 4°C to remove debris, and the supernatant was collected and stored at -80°C until used. Protein concentrations were determined using the Bio-Rad assay kit (Hercules, CA, USA) according to manufacturer’s recommendations. A total of 60 μg protein was boiled in sample buffer for 5 minutes, and then proteins were separated by sodium dodecyl sulfate polyacrylamide gel electrophoresis (SDS-PAGE) using a 10% gel (running buffer: 25 mM Tris, 250 mM glycine, 0.1% SDS). Subsequent to SDS-PAGE, proteins were transferred overnight to nitrocellulose transfer membranes (BioRad), which were then blocked in 5% non-fat milk in Tris-buffered saline-Tween (TBST: 25 mM Tris-base, 0.02% KCl, 137 mM NaCl, 0.05% Tween 20) for 1 hour. Immunoblotting was performed by incubation with primary antibodies at 25°C for 2 hours (Table 1). GAPDH (polyclonal, 1:10000, GTX100118; GeneTex, CA, USA) was used as an endogenous protein for normalization. Blots were then washed and incubated with a 1:2000 dilution of horseradish peroxidase (HRP)-conjugated secondary antibody (Jackson, West Grove, Pennsylvania, USA), followed by three washes with TBST. Enhanced chemiluminescent HRP substrate (Pierce, Rockford, IL, USA) was used for detection according to manufacturer’s description.

**Table 1:** Description of western blot and immunohistochemical antibodies.

Antibody	Vendor	Clone	WB (dilution)	IHC (dilution)
Cullin 4A	Abcam	EPR3198	1:1,000	1:200
IRS2	Abcam	EP976Y	1:100,000	1:150
TFDP1	Abcam	Polyclonal	1:1,000	1:100

### Transient cell transfection and immunofluorescence detection

The IRS2 overexpression vector (pCMV Entry-IRS2-Myc-DDK, Cat. No. RC212413) and specific siRNAs targeting *IRS2*, *CUL4A* and *TFDP1* were purchased from Origene (Origene, MD, USA). Cells were transiently transfected using Lipofectamine 3000 (Invitrogen, NY, USA) according to the manufacturer's recommendations. Briefly, 2.5 × 10^5^ cells were plated in six-well plates one day before transfection. Lipofectamine 3000, expression vector and siRNA were diluted in Opti-MEM medium (Invitrogen). A total of 250 μl mixtures were incubated for 5 minutes at room temperature. Transfection complexes were added to the cells incubated in a humidified atmosphere, 5% CO_2_ at 37°C, for 48 hours. Expression levels of the target genes were evaluated by Western blotting. Cellular proliferation was studied by analyzing proliferating cell nuclear antigen (PCNA; 1;2000, Origene). Total cell numbers were calculated by staining cells with trypan blue and manual counting at a 400 × magnification by Neubauer hemacytometer (Marienfeld, Baden-Württemberg, Germany). Experiments were conducted in triplicate. The transfection efficiency was checked by Western blotting using anti-DDK antibody (1:1000; Origene) and immunofluorescence detection of IRS2. Cells growing on cover glass slides were washed several times with PBS, fixed with methanol for 10 min at -20°C, and then blocked with 1% skim milk in PBS. The slides were incubated with IRS2 primary antibody (1:200; Abcam) at room temperature for 1.5 hours, washed with three changes of PBS for 5 min, and then incubated with a 1:250 dilution of fluorescence-labeled goat anti-rabbit secondary antibody (Jackson) at 37°C for 0.5 hour. After additional washing steps, cells were counterstained with 4',6-diamidino-2-phenylindole (DAPI; Sigma-Aldrich, Munich, Germany) in a concentration of 100 ng/ml for 10 min, and mounted on microscope slides with ProLong antifade mounting medium (ThermoFisher, MA, USA). The fluorescence signals in transfected cells were detected with a fluorescence microscope (BX51, Olympus, Tokyo, Japan) and images were taken with an Olympus DP70 digital camera.

### Cell migration and invasion assays

Cell migration and invasion were assessed by using 24-Transwell® plates. Transwells with 8-μm pores were coated with 0.1 mL of the diluted Matrigel Matrix coating solution (Corning, NY, USA) for the invasion assay, or left uncoated for the migration assay. RPMI (800 μl) containing 10% FBS was placed in the bottom wells of the chamber, and 200 μl of cell suspension (2 × 10^4^ cells for migration and 3 × 10^4^ cells for invasion) were added to the top wells of the chamber. Subsequent to incubation in a humidified atmosphere, 5% CO_2_ at 37°C, for 24 hours, migrating or invading cells present on the lower surface of the filter were fixed in 100% methanol, and stained with Giemsa staining solution (Merck, Germany). The average cell mobility was determined by counting three random high-powered fields at ×100. Three independent experiments were performed for both invasion and migration assays.

### Immunohistochemical analysis

Immunohistochemical (IHC) stains were performed as previously described [15]. Primary antibodies against CUL4A, IRS2, and TFDP1 were used and followed by the PicTureTM-Plus kit (ZYMED®; 2nd Generation Polymer Detection System, San Francisco, CA, USA) (Table 1). A total of 86 formalin-fixed, paraffin-embedded iCCA tissue samples were used for tissue microarray construction, as described by Huang et al [16]. The RBE cell line was used as a positive control. To determine the reactivity of the immunostains, normal bile ducts from 10 representative non-tumor sections were selected for comparison. Slides were evaluated by a pathologist (TTL), blind to clinicopathological data. The labeling intensity was classified as negative, weak, moderate, and strong. The percentages of tumor cells with moderate or strong cytoplasmic immunoreactivity for IRS2 and nuclear immunoreactivities for CUL4A and TFDP1 were recorded as expression index in 5% increment. Only cases containing two or more analyzable cores were scored, and scores of multiple cores from the same patient were averaged to obtain a mean labeling index. Whole sections were performed for IHC stains in cases with non-informative tissue cores (no tumor or analyzable cores < 2).

### Statistical analysis

All statistical analyses were performed using Statistical Product and Service Solutions (SPSS) for windows 17.0 software (SPSS Inc. Chicago, IL). The correlation between CN and protein expression was evaluated by Pearson’s correlation test. Comparisons between different two groups were undertaken using the Student’s *t*-test. The significance of association between CN alterations and histopathological variables was determined by Chi-square and Fisher exact test. Overall survival was calculated from the date of diagnosis to death as a result of all causes. Disease-free survival (DFS) was computed from the time of surgery to recurrence in the liver or distant metastasis. The Kaplan–Meier method was used for univariate survival analysis, and the difference between survival curves was tested by a log-rank test. In a stepwise forward fashion, parameters with *P* values < 0.05 at the univariate level were entered into a Cox regression model to analyze their relative prognostic importance. However, the vascular invasion and tumor number components of the 7^th^ AJCC staging system were not introduced into the multivariate analyses. For all analyses, two-sided tests of significance were used with *P* < 0.05 considered significant.

## Results

### CN alterations correlated with protein expression levels and the mobility potential in the cell lines of iCCA

**Fig 1:**
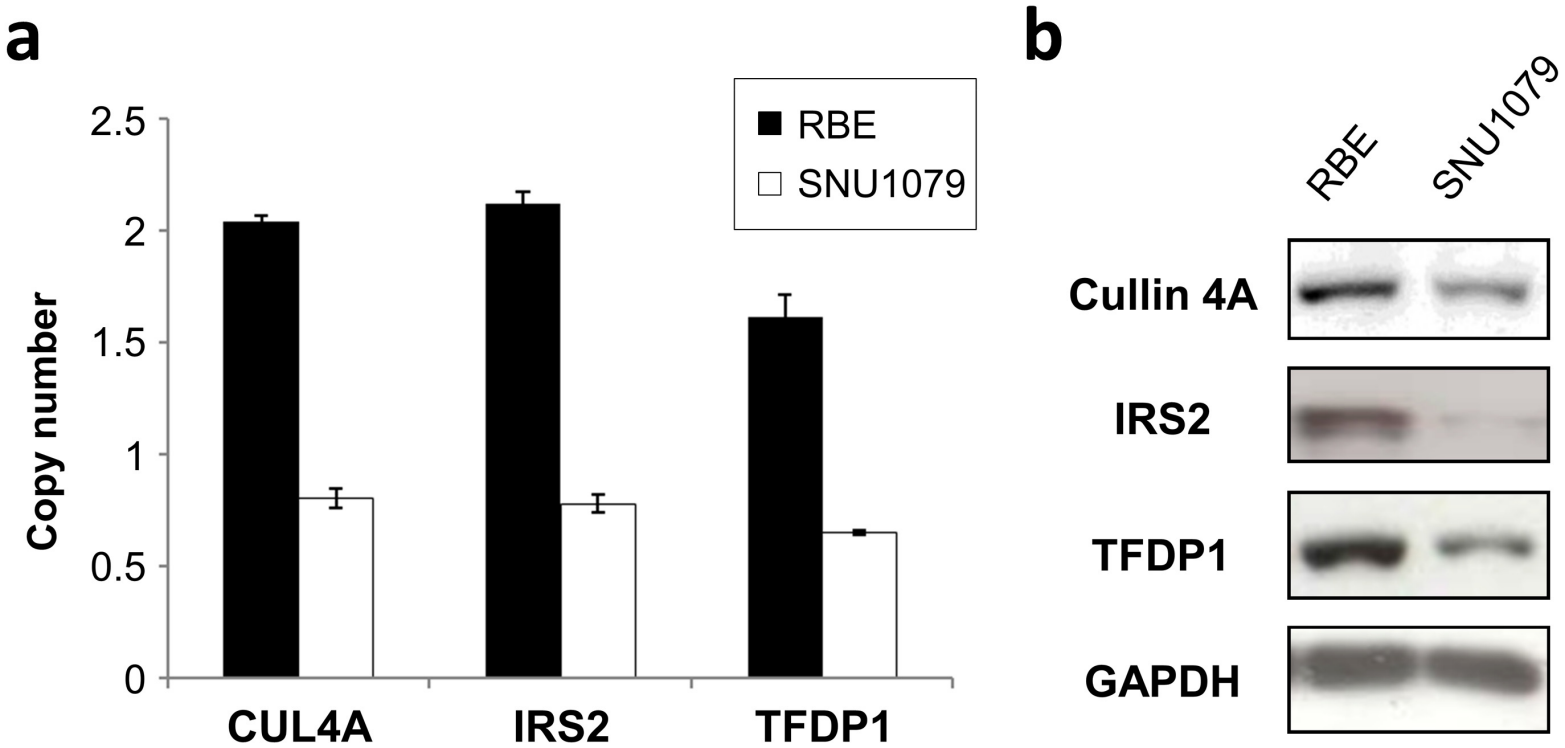
Cell line copy number and protein expression. (a) Compared to the RBE cell line, copy numbers in the SNU1079 cell line was decreased. Data represent means ± standard deviations for three independent experiments. (b) The result was correlated with the protein expression demonstrated by Western blot analysis.

**Fig 2:**
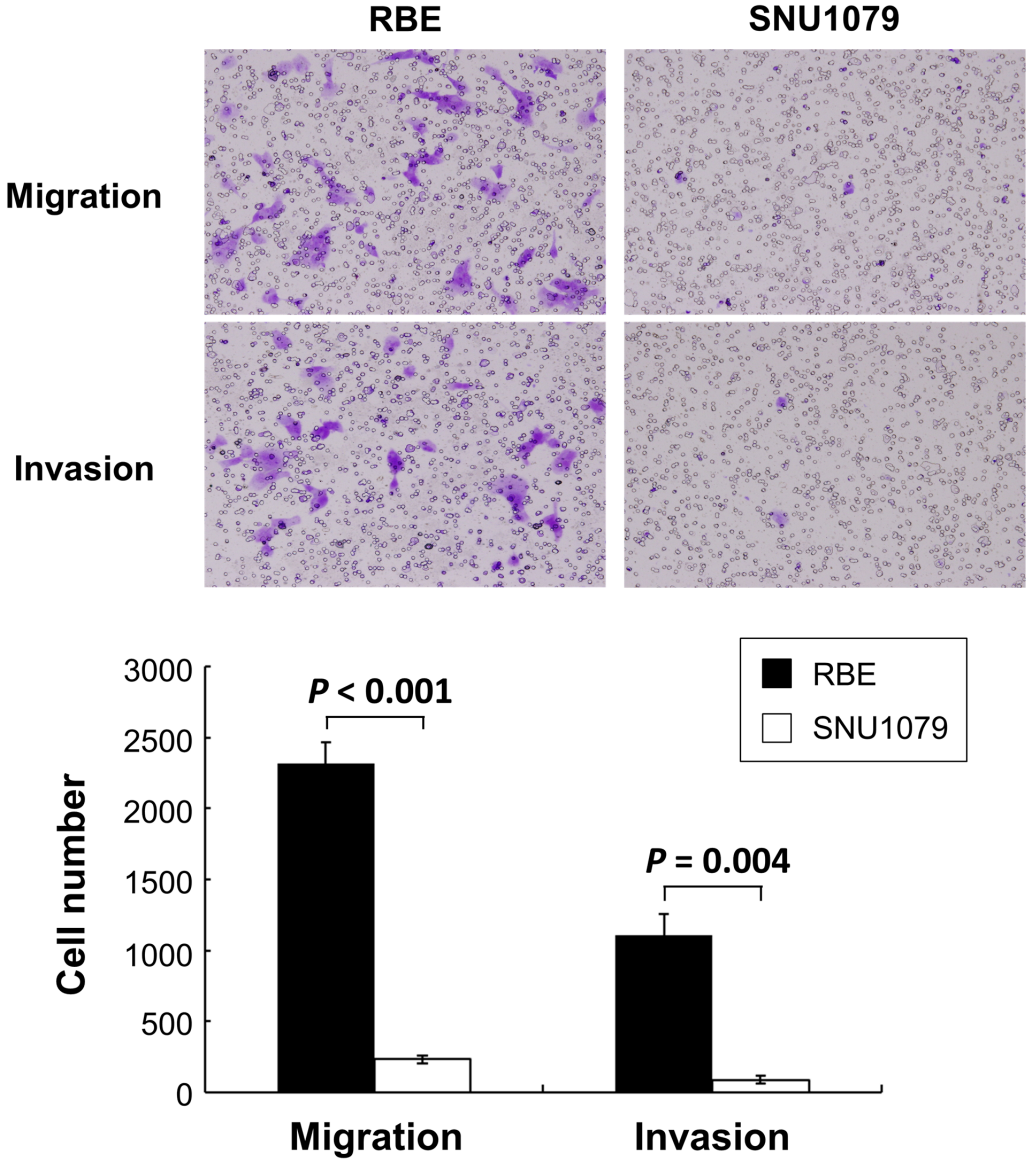
Cell line mobility potential. The RBE cell line demonstrated an increased number of migratory and invasive cells on the bottom of the wells. In contrast, the SNU1079 cell line containing a deletion of target genes displayed a significant decrease to mobility potential. Data represent means ± standard deviations for three independent experiments.

We first examined CN alterations, protein expression levels and the mobility potential in two cell lines. *CUL4A*, *IRS2*, and *TFDP1* were deleted in SNU1079 cells in contrast to normal genomic DNA derived from peripheral blood lymphocytes. The SNU1079 cell line with deleted genes exhibited decreased protein expression levels, as opposed to the RBE cell line that did not have remarkable CN alterations (Fig 1). Next, we examined the mobility potential of the cell lines, which influences local recurrence and distant metastasis. Compared to RBE cells, SNU1079 cells containing deletions of the target genes had significantly lower numbers of migratory and invasive cells (Fig 2).

### Manipulation of the target genes affecting migratory and invasive capacities of iCCA cells in vitro

**Fig 3:**
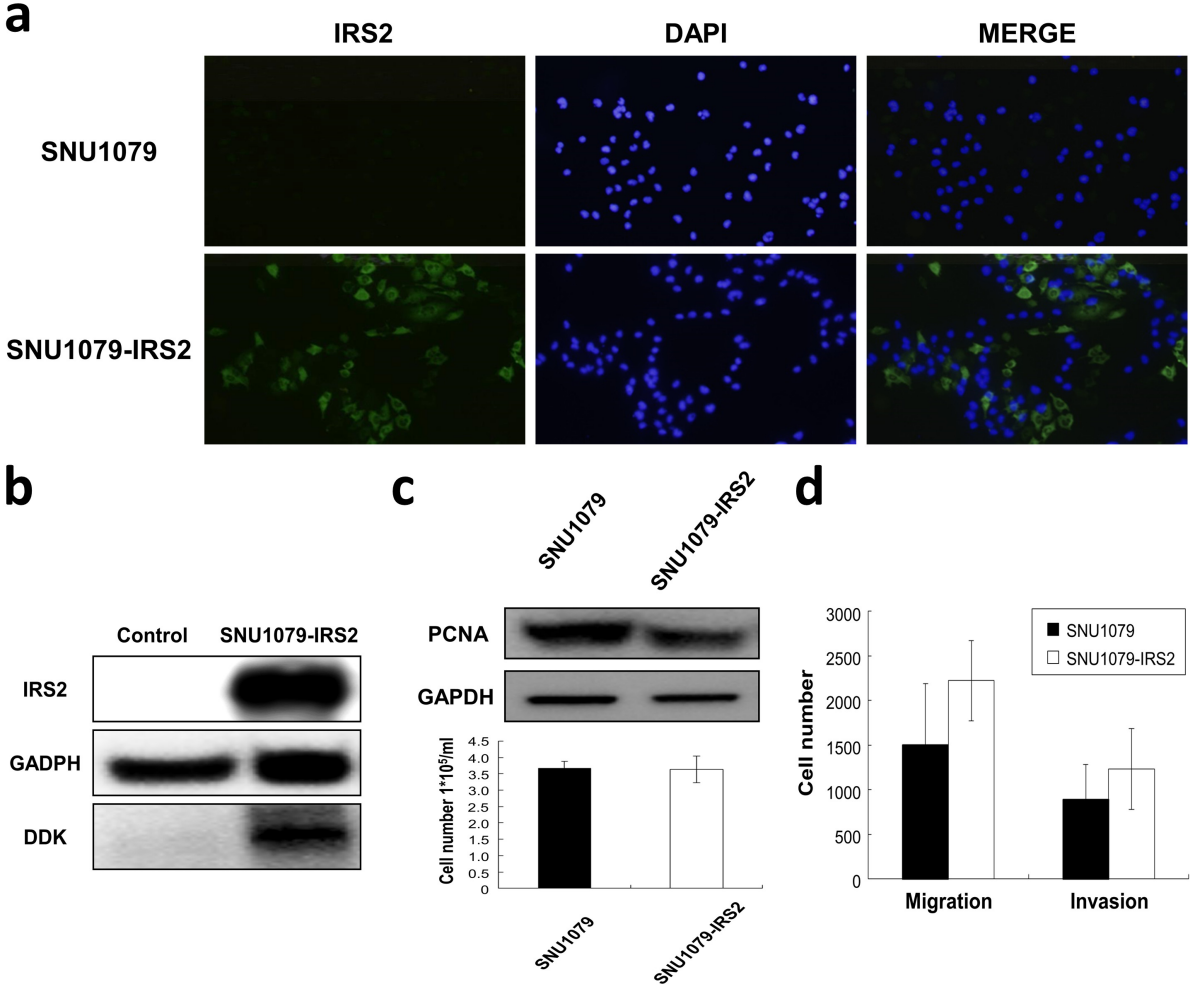
Enforced expression of IRS2 in iCCA cells. (a) SNU1079 cells were transiently transfected with expression vector (SNU1079-IRS2). After 48 hours, expression of *IRS2* was determined by immunofluorescence detection of IRS2 protein (left panel, green) and DAPI (middle panel, blue). MERGE contains the combined image of both GFP and DAPI staining (right panel). Percentage of IRS2-positive SNU1079 cells was normalized to DAPI stained nuclei. Images were obtained at a magnification of × 100. (b) Total cell lysates were analyzed for IRS2 protein levels by Western blotting. Anti-DDK tag antibody was used to reveal the extent of IRS2 overexpression. (c) The effect of IRS2 overexpression on growth potential was determined by Western blotting using anti-PCNA antibody and manual counting of total cell numbers. (d) SNU1079-IRS2 cells were subjected to invasion and migration assays, and showed a greater degree of migration and invasion, compared with control cells. Graphs represent data from one of three independent experiments with similar results. Data represent means ± standard deviations for three independent experiments.

**Fig 4:**
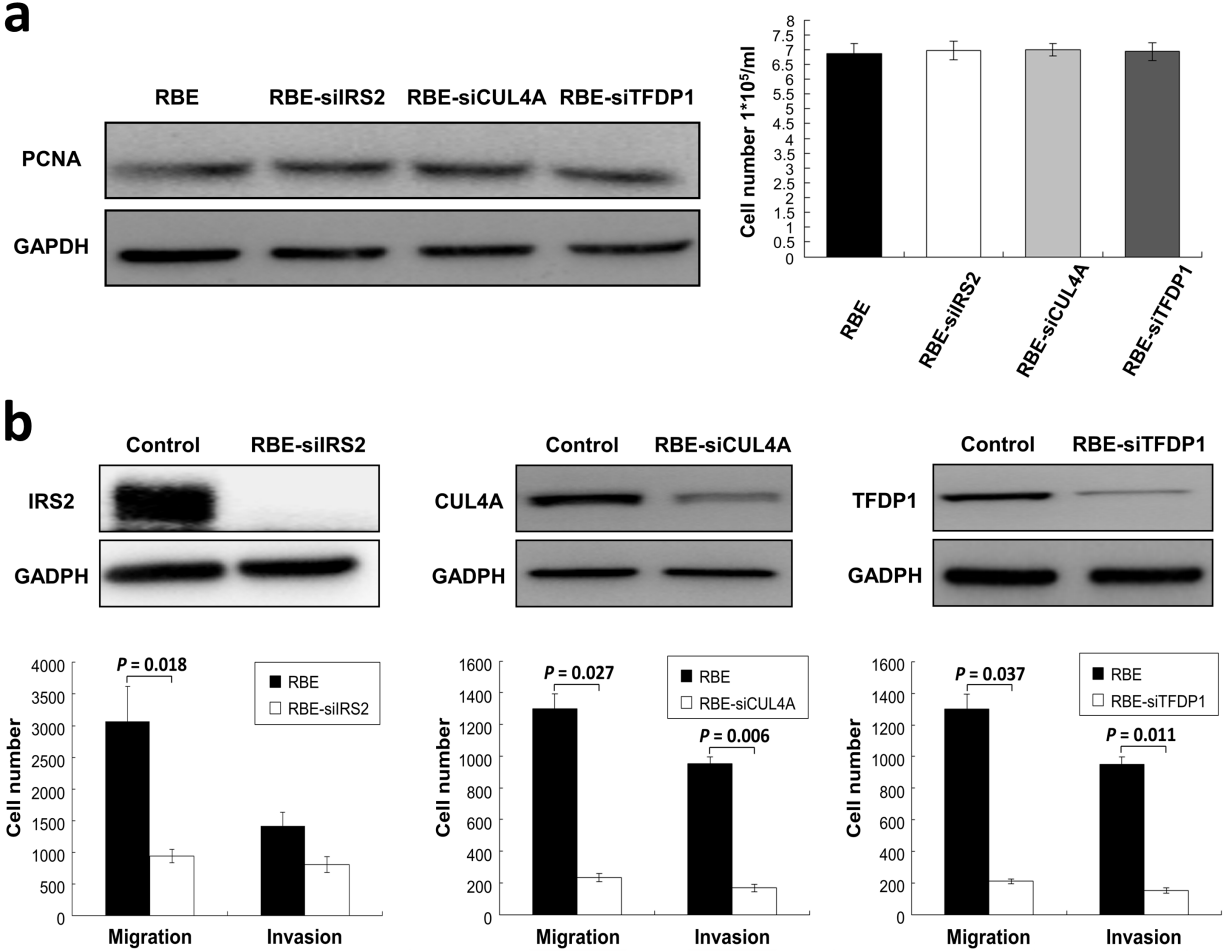
Silencing of *IRS2*, *CUL4A* and *TFDP1* in iCCA cells. (a) RBE cells were transiently transfected with siRNAs (RBE-siIRS2, RBE-siCUL4A and RBE-siTFDP1). The effect of gene expression after manipulation on growth potential was determined by Western blotting using anti-PCNA antibody (left) and manual counting of total cell numbers (right). (b) Silencing of the target genes, which were confirmed by Western blotting, dramatically reduced the mobility potential of RBE cells. Data represent means ± standard deviations for three independent experiments.

In order to test the oncogenic activity of the target genes in cell lines, we introduced *IRS2* in SNUI079 cells (designated as SNU1079-IRS2), and silenced each target gene in RBE cells (designated as RBE-siIRS2, RBE-siCUL4A and RBE-siTFDP1). Immunofluorescence microscopy (Fig 3A) and Western blot (Fig 3B) analyses demonstrated the effectively up-regulated expression of IRS2 without significant impact on the cellular proliferation (Fig 3C). SNU1079-IRS2 cells had higher numbers of migratory and invasive cells compared to their control cells (Fig 3D). While knockdown of the target genes in RBE cells, which showed no significant impacts on the cellular proliferation due to gene silencing (Fig 4A), RBE-siIRS2 cells showed a trend toward inhibition of the mobility potential and a significant lower degree of migration (*P* = 0.018), and both RBE-siCUL4A and RBE-siTFDP1cells had significant lower migratory (*P* = 0.027 and *P* = 0.037, respectively) and invasive (*P* = 0.006 and *P* = 0.011, respectively) capacities than control cells (Fig 4B).

### Amplification of genes at 13q34 associated with poor DFS and correlated with protein expression levels in tumor samples of iCCA

**Fig 5:**
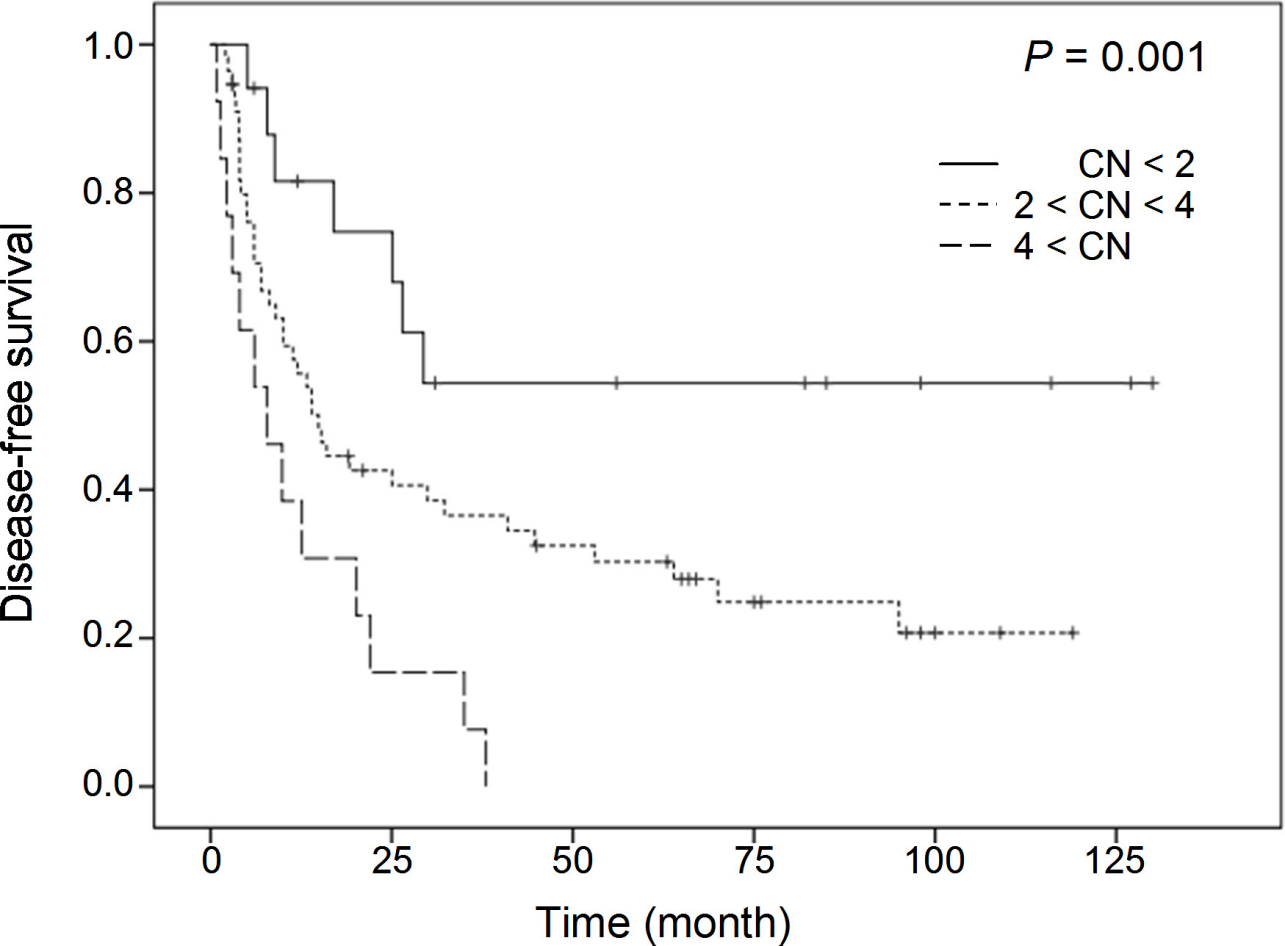
Kaplan-Meier survival curves for patients categorized by gene copy numbers. Statistical significance was observed among groups. (CN < 2: copy numbers less than two for all three markers of the 3-marker combination; 2 < CN < 4: copy numbers between two and four for at least one of three markers; 4 < CN: copy numbers more than four for all three markers)

**Fig 6:**
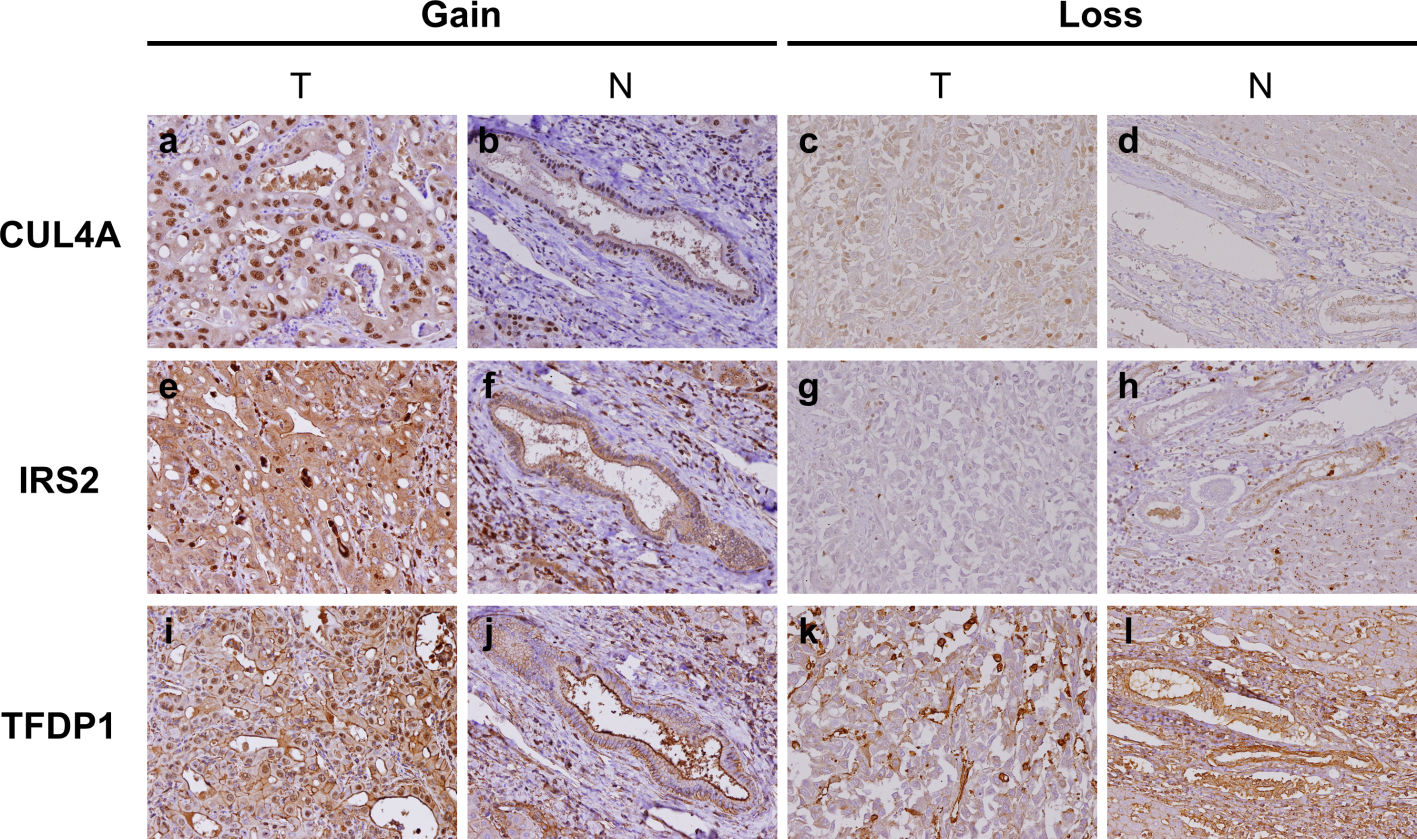
Immunohistochemical analysis in tumors (T) of intrahepatic cholangiocarcinoma and normal bile ductules (N) of the adjacent portal tracts. Positive nuclear staining was observed for CUL 4A and TFDP1, and cytoplasmic staining for IRS2 in tumor cells with amplification of target genes (a, e, i), compared with normal bile ductules in the adjacent portal tracts (b, f, j). Loss of expression for CUL 4A, IRS2, and TFDP1 (c, g, k) was observed in tumor cells containing a deletion of target genes, in contrast to their normal counterparts (d, h, l). Original magnification: × 200.

Table 2 and Table 3 summarize clinicopathologic characteristics and CN alterations of three target genes, respectively. CN detection failed for three (3.5%) of the 86 *IRS2* cases probably due to DNA degradation. Kaplan-Meier univariate survival analysis revealed that amplification of *CUL4A*, *IRS2*, and *TFDP1* was associated with poor DFS. A 3-marker combination consisting of *CUL4A*, *IRS2*, and *TFDP1* also showed a significant difference in DFS. When tumors showed a CN higher than four for all three markers with more than 4 copies (n = 12, 13.9%, median 7.8 months), DFS was shortened in comparison to cases with a CN between two and four for at least one of three markers with more than two but less than four copies (n = 57, 66.3%, median 14.9 months) or lower than two for all three markers with less than two copies (n = 17, 19.8%, median 17.0 months, *P* = 0.001, Fig 5). Multivariate Cox proportional hazards regression analysis revealed that amplification of the 3-marker combination, consisting of *CUL4A*, *IRS2*, and *TFDP1* with all copies higher than four might be an independent adverse prognosticator for DFS (Table 4). To determine whether the three amplified genes examined were also overexpressed, we performed IHC analyses to compare gene CN and protein expression. The results showed positive correlation with statistical significance in *IRS2* and *CUL4A* (r = 0.368; *P* = 0.001 and r = 0.401; *P* < 0.001, respectively) and marginal significance in *TFDP1* (r = 0.188; *P* = 0.08). A strong nuclear staining of CUL4A and TFDP1, and increased cytoplasmic staining of IRS2 were identified in tumors with amplification, compared to normal bile ducts and cases with a deletion of target genes (Fig 6).

**Table 2:** Results of univariate long-rank analysis of prognostic factors for overall survival and disease-free survival.

Parameters	No. of patients	Overall survival		Disease-free survival	
		No. of events	P value	No. of events	P value
Age, years					
≤ 60	49	30	0.221	34	0.821
> 60	37	26		26	
Gender					
Male	48	35	0.312	33	0.522
Female	38	21		27	
Gross pattern					
MF	52	30	0.027*	36	0.448
MF + PI	33	26		23	
Tumor N					
Solitary	69	43	0.052	45	0.008*
Multiple	16	12		15	
Tumor size					
≤ 5 cm	46	27	0.054	25	0.001*
> 5 cm	40	27		33	
Margin					
≤ 1 cm	59	41	0.049*	44	0.019*
> 1 cm	25	14		15	
Necrosis					
≤ 10%	62	39	0.274	41	0.169
> 10%	24	17		19	
VI					
No	52	32	0.408	31	0.02*
Yes	34	24		29	
NI					
No	55	30	0.004*	35	0.058
Yes	31	26		25	
H grade					
I	26	17	0.716	14	0.069
II + III	60	39		46	
pT					
T1	27	14	0.043*	15	0.018*
T2—T4	59	42		45	
LN					
No	33	20	0.233	23	0.507
Yes	9	7		7	
Stage					
I	25	13	0.032*	14	0.028*
II + III + IV	61	43		46	

M, mass-forming type; PI, periductal infiltrating type; N, number; VI, vascular invasion; NI, neural invasion; H, histology; pT, tumor stage; LN, lymph node metastasis

*Statistically significant

**Table 3:** Copy number changes of target genes at 13q34.

Genes	Total cases	CN	Q > 4
	*n*	mean (range)	*n* (%)
*IRS2*	83	4.26 (0.49–29.20)	28 (33.7)
*TFDP1*	86	3.92 (0.38–24.37)	24 (27.9)
*CUL4A*	86	3.04 (0.24–10.00)	16 (18.6)

CN: copy number; Q: copy number copies

**Table 4:** Independent predictive factors of disease-free survival by multivariate analysis.

Variable	A 3-marker combination (*IRS2*, *TFDP1*, *CUL4A*)		
	Hazard Ratio	95% CI	*P*
Tumor size ≤ 5 cm vs > 5 cm	2.04	1.19 to 3.50	0.010
Resection margin ≤ 1 cm vs > 1 cm	2.32	1.23 to 4.38	0.010
Stage I vs II & III & IV	1.95	1.02 to 3.73	0.045
Copy number < 4 vs > 4	2.69	1.23 to 5.88	0.013

## Discussion

In this study we characterized CN changes of three target genes mapping to 13q34 in iCCA cases. Amplification of the target genes may be associated with protein expression and influence tumor migration and invasion. By introducing and silencing the target genes, the mobility abilities of cell lines were promoted and inhibited, respectively. The manipulation of the target genes affected migratory and invasive capacities of iCCA cells. We then delineated the frequencies of gene amplifications in tumor samples. The correlations that we identified suggest that amplifications of *CUL4A*, *IRS2*, and *TFDP1* are adverse prognosticators of DFS. To the best of our knowledge, this is the first study to elucidate the oncogenic role of 13q34 in iCCA.

Although fluorescent in situ hybridization (FISH) has been the gold standard technique to detect gene CN, qPCR could provide an alternative and powerful method [17,18]. qPCR is easy to perform, less expensive than FISH, and can be used to analyze a large area of tumors, thus minimizing CN deviation due to tumor heterogeneity. Jacquemier et al. showed 95% and 93% of concordance between FISH and qPCR with sensitivity ranging from 89% to 80%, based on an examination of the HER2/ chromosome 17 centromere ratio or HER2 CN, respectively [17]. In addition, determination of gene CN by qPCR is objective and reliable. In a previous whole-genome study, we found that amplification of 13q34 was identified in five (15.6%) of 32 fresh iCCA samples [19], similar to the frequency of cases (13.9%) with a CN higher than four for genes *CUL4A*, *IRS2*, and *TFDP1* found in the present study. A 3-marker combination consisting of *CUL4A*, *IRS2*, and *TFDP1* is useful for determining 13q34 amplification by qPCR.

*IRS2* is a candidate driver oncogene that is frequently amplified in colorectal cancer and significantly positively correlated with *IRS2* mRNA expression [10,20–22]. Overexpression of IRS2 increases colorectal cell adhesion, similar to observations made for breast cancer [10,23,24]. IRS2 is an adaptor in the insulin and insulin-like growth receptor signaling cascades [25]. Overexpression of IRS2 may result in AKT phosphorylation, thus promoting tumor progression [10]. In the present study, we found manipulation of *IRS2* affected migratory and invasive capacities of iCCA cell lines. The result indicates *IRS2* may be a potential therapeutic target for iCCA.

Amplification of *TFDP1* contributing to gene overexpression was reported in HCC, breast cancer, and lung cancer [9,8,7]. *TFDP1* encodes transcriptional factor DP-1, which is a heterodimerization partner for members of E2F. The E2F/DP-1 complex regulates expression of cell cycle promoters [26]. In HCC, overexpression of *TFDP1* may contribute to tumorigenesis by up-regulating expression of *CCNE1* that encodes for cyclin E of the cell cycle G1/S transition, resulting in tumor progression [7,27]. Down-regulation of *TFDP1* reduces cell growth as demonstrated in HCC and lung cancer cell lines [9,27]. Melchor et al. established that breast tumors overexpressing TFDP1 were significantly associated with 13q34 amplification, and had high expression of p16, cyclin E, and cyclin B1 [8].

Similar to *TFDP1*, amplification of *CUL4A* DNA is also correlated with gene expression [8,7]. CUL4A, a member of the cullin family of proteins comprising the multifunctional ubiquitin-protein ligase E3 complex, is associated with the ubiquitination of tumor suppressor genes [28–30]. Overexpression of CUL4A is associated with tumor proliferation, progression, and metastasis [8,12]. Wang et al. further demonstrated that CUL4A overexpression increases the epithelial-mesenchymal transition and promotes the metastatic capacity in breast cancer [12].

Our findings, along with the previous research [8–10,12,23,24,27], suggest the importance of the target genes as targets for 13q34 amplification and biological implications for tumor progression. Theses target genes could be potential targets for developing anti-cancer therapeutics. In addition, it was shown that CUL4A levels can be used as a biomarker for predicting cancer cell sensitivity to a particular therapeutic [31,32]. Although a larger cohort study would be needed to declare the prevalence of 13q34 amplification, this genomic aberration may be used both as a clinical marker and as a therapeutic target in iCCA.

In summary, the present study highlights the diagnostic potential of 13q34 amplification by targeting *CUL4A*, *IRS2*, and *TFDP1* in iCCA. The 3-marker combination is an indicator of DFS, independent of resection margin and tumor stage. Patients exhibiting amplification of target genes have an adverse DFS. Further research into the mobility-associated molecular pathogenesis of target genes may provide new directions for targeted therapy.
